# Nerve root herniation with entrapment in the facet joint gap after lumbar decompression surgery: a case presentation

**DOI:** 10.1186/s12891-021-04601-1

**Published:** 2021-08-27

**Authors:** Branko Popadic, Florian Scheichel, Melanie Themesl, Ingo Decristoforo, Camillo Sherif, Franz Marhold

**Affiliations:** 1 Karl Landsteiner University of Health Sciences, Dr. Karl-Dorrek-Straße 30, 3500 Krems, Austria; 2 Department of Neurosurgery, University Hospital St. Pölten, Dunant-Platz 1, 3100 St. Pölten, Austria

**Keywords:** Spine surgery, Complication, Nerve root herniation, Entrapment, Case report

## Abstract

**Background:**

An incidental dural tear is a well-known complication during spine surgery. A rare consequence is a postoperative nerve root herniation. The purpose of this report is to describe a case of such a herniation with entrapment in the facet gap joint and to present the first MR images of this rare surgical complication.

**Case presentation:**

We report a case of a patient who underwent lumbar decompression surgery and afterwards suffered a sudden intractable sciatica. Postoperative MRI showed a new facet joint gap effusion. During revision surgery an entrapped nerve root was found in the facet joint gap. In retrospective, the herniated nerve root is visible on postoperative MRI.

**Conclusion:**

This case report highlights a rare complication during spine surgery. This finding is important as signs suggestive for nerve root herniation can easily be overlooked on MRI. Furthermore, this represents the first MRI documentation of this complication.

## Background

A well-known complication during lumbar spine surgery is an incidental dural tear. Depending on the type of surgical procedure the rates in the literature range from 1 to 17% [1]. Less common are intraoperative unrecognized or postoperative spontaneous dural tears leading to subsequent symptoms with reports of 0.28% in spine surgery [2]. A nerve root herniation through such an unrecognized or spontaneous dural tear with nerve entrapment is a rare consequence and the literature consist mostly of case reports [3, 4]. So far, a postoperative nerve root herniation with entrapment in the facet gap joint has been reported only once and dates back to the pre-magnetic resonance imaging (MRI) area [5].

We describe an exceptional case of such a herniation and present the first MR images of this rare surgical complication.

## Case presentation

**Fig. 1:**
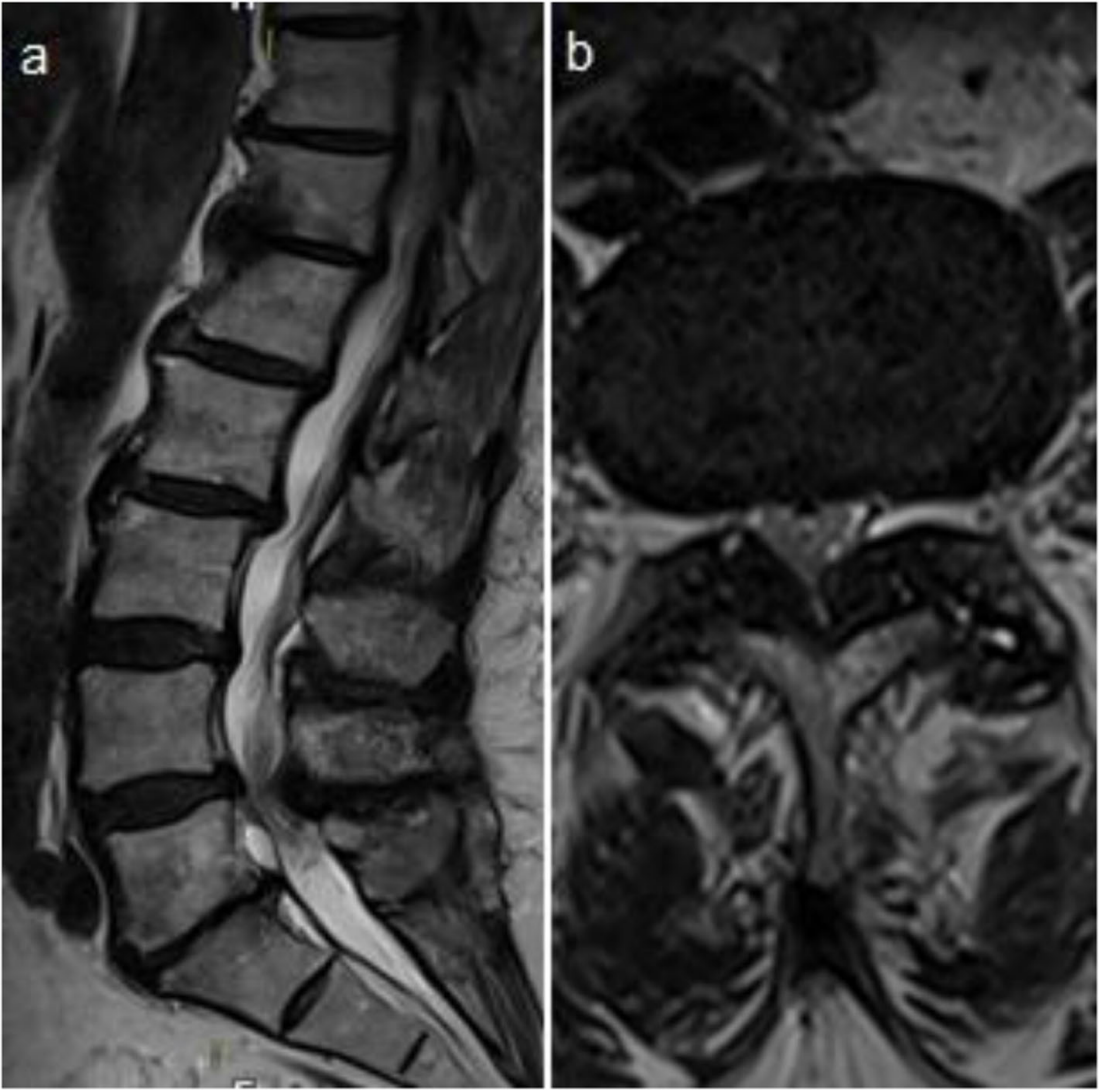
Preoperative sagittal (**a**) and axial (**b**) T2-weighted MRI showing a spinal stenosis at the level of L4/5. Note, that there is no facet gap joint preoperatively

A 64-year old woman presented with neurogenic claudication and a maximum walking distance of 50 m. She described a radiating pain from the gluteal region to the lateral legs on both sides with a predominance for the left side, with a symptom onset approximately 1 year ago. Physical examination revealed intact motor function and decreased deep tendon reflexes on the left side. Medical history consisted of a discectomy L5/S1 on the right side 13 years ago. MRI revealed a lumbar spinal stenosis at the level of L4/5 (Fig. 1). Patient underwent a standard microsurgical decompression L4/5 on the left side, including an over the top undercutting to the right side. During surgery, no dural tear was noted. On the first postoperative day she was mobilised and described significant improvement of the preoperative pain.

**Fig. 2:**
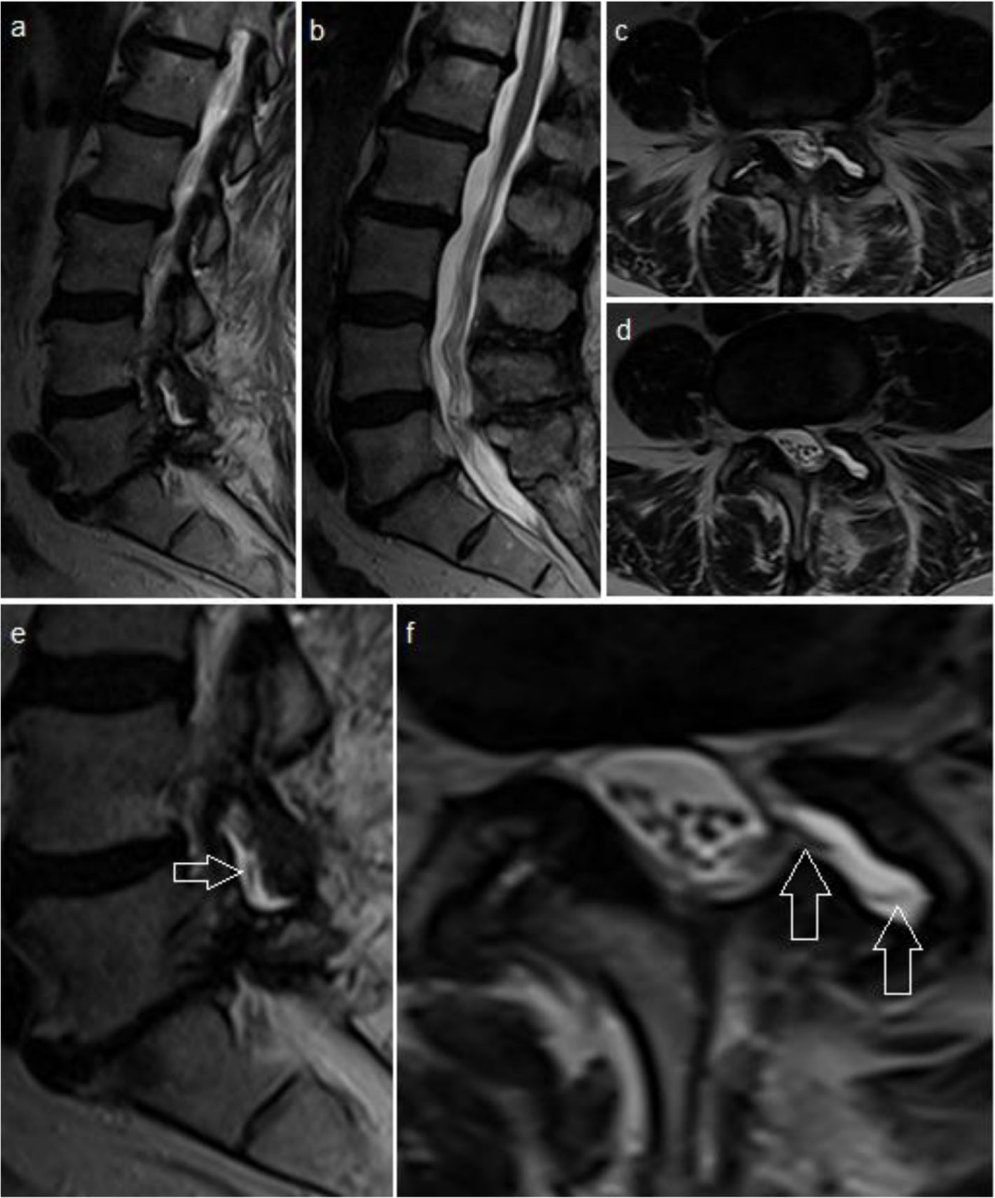
MRI after decompression surgery and sudden sciatica onset: Sagittal (**a-b)** and axial (**c-d**) T2 weighted images showing no hematoma or intraspinal compression. An effusion of the facet joint is noted. Zoomed sagittal (**e**) and axial (**f**) image shows herniation of a nerve root (white arrows) into the facet joint gap

**Fig. 3:**
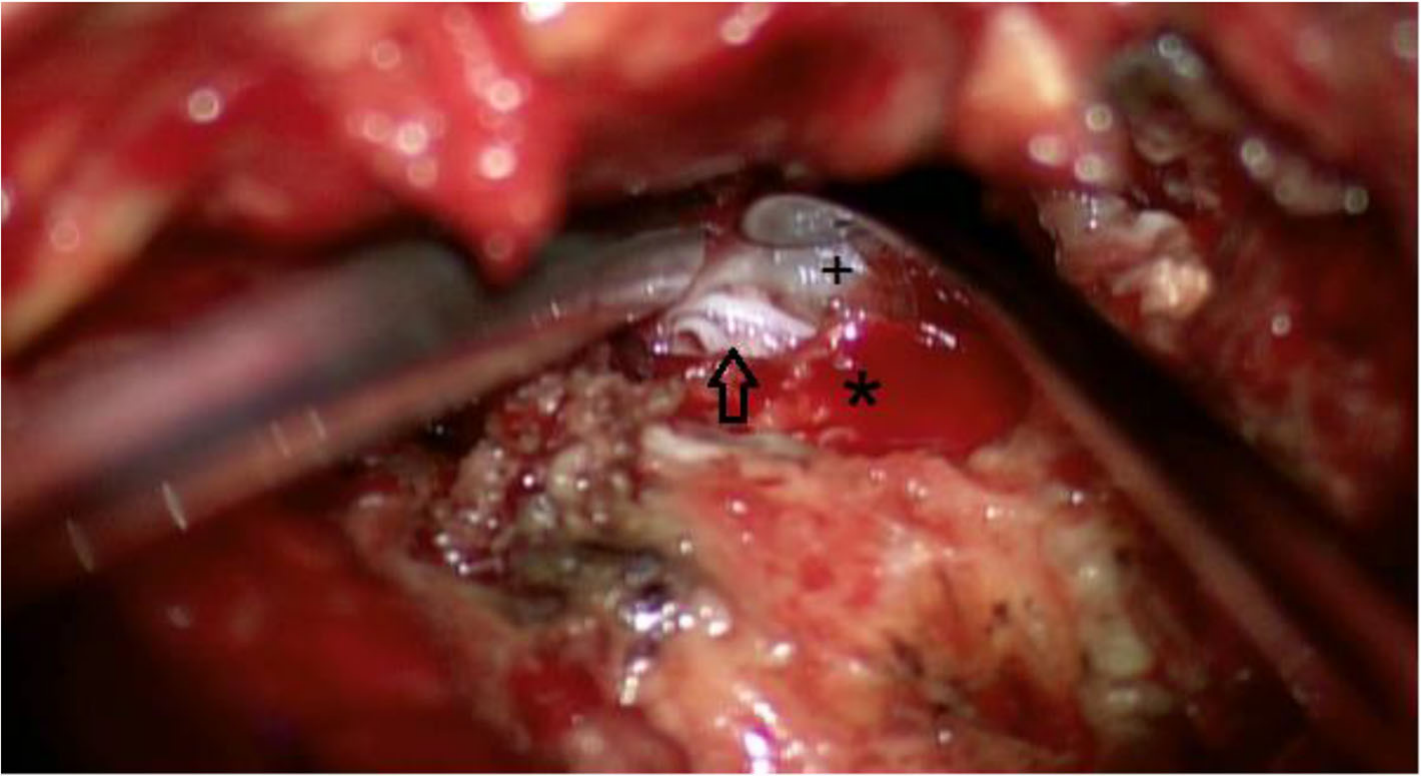
Intraoperative photograph during revision surgery L4/5 from the left side. The dura is visible under the curette (plus sign), as well as the remaining part of the left facet joint L4/5 (asterisk). A dural tear is revealed with a herniated nerve root (black arrow), which was entrapped into the facet joint gap

Two days later, during a turning movement in bed, she experienced a sudden sciatica radiating in her left leg. The intense pain was not responding to any analgesics and was only tolerated in a standing position leaning on the right leg. After frustrating attempts to stay in the horizontal position despite intravenous opioid administration, she was put under general anaesthesia for an MRI scan. The imaging revealed no hematoma or significant intraspinal compression. However, an unclear facet joint effusion L4/5 on the left side was apparent, which was not present preoperatively (Fig. 2). The decision for exploratory revision surgery was therefore manly based on her clinical presentation as at this point the meaning of this effusion was unclear. In early stages of revision surgery cerebrospinal fluid (CSF) leakage was noted and after careful dissection, a nerve root herniation through a small lateral dural tear with entrapment in the facet joint gap was discovered (Fig. 3). The herniated root was repositioned and the dural defect was repaired using stitches in a watertight fashion and a sealant matrix. Postoperatively the patient’s symptoms disappeared, she was mobilised after 2 days using a lumbar brace and the further clinical course was uneventful. As she did not develop any further clinical signs of potential segmental instability, fusion surgery was not deemed necessary.

## Discussion and conclusion

Recurrence of radicular pain after microsurgical lumbar decompression surgery can have many reasons. Epidural hematoma, disc herniation or insufficient decompression are frequent findings on postoperative imaging in symptomatic patients. In cases of incidental intraoperative dural tears, physicians are usually attentive for symptoms of CSF leaks like postural headache with nausea etc. during the postoperative period [6]. However, a less known complication of such a dural tear represents a postoperative nerve root herniation. Several authors have described cases of nerve root herniations with entrapments in the intervertebral disc space [3, 4, 7]. The clinical course of their cases showed a close resemblance to our patient with a temporary improvement after surgery and a sudden onset of radiating pain showing little to no improvement to analgesics.

The postoperative imaging in our case showed no hematoma or compression of the nerve root and the decision for exploratory revision surgery was based mainly on the clinical presentation of the patient. Furthermore, MRI revealed a distinct facet joint effusion L4/5 on the left side, which was not visible on preoperative imaging. Intraoperatively, a herniated nerve root was found in this facet joint gap. In retrospect, this herniated nerve root was visible on MRI as a thin structure exiting the dura and entering the facet joint gap (detailed description in Fig. 2). Bae et al. [4] reported a similar experience in cases with postoperative nerve root herniation into the disc space. They described a CSF signal in the disc space as well as a visible displaced nerve root in their cases on retrospective review of the images.

These conclusions are important as physicians focus their search on compressive lesions in a postoperative MRI of a symptomatic patient. Findings that are suggestive for a dural defect or nerve root herniation can easily be overlooked.

To the best of our knowledge, this is the second report about this unusual complication and the first MRI documentation. In 1995, Nishi et al. [5] described a patient with a postoperative herniation and entrapment of the S1 nerve root into the facet joint after a hemilaminectomy. They performed myelography and computed tomography-myelography with pathological findings. Nevertheless, the clinical course and intraoperative findings show close similarities to our patient.

Nerve root herniation with entrapment represents a rare complication after lumbar spine surgery. In patients with sudden postoperative recurrence of intractable radiating pain and postoperative MR imaging without hematoma or nerve compression, physicians should consider this as a possibility. In these patients, special attention must be diverted for findings suggestive of nerve root herniation as they can be easily overlooked.

## Data Availability

Not applicable.

## Abbreviations

MRI: Magnetic resonance imaging
MR: Magnetic resonance
CSF: Cerebrospinal fluid
